# Role of public-private partnerships in achieving UNAIDS HIV treatment targets

**DOI:** 10.1186/s12913-018-3744-z

**Published:** 2019-01-18

**Authors:** Ritu Shrivastava, Peter N. Fonjungo, Yenew Kebede, Rajendra Bhimaraj, Shabnam Zavahir, Christina Mwangi, Renuka Gadde, Heather Alexander, Patricia L. Riley, Andrea Kim, John N. Nkengasong

**Affiliations:** 1 International Laboratory Branch, Division of Global HIV/AIDS, Center for Global Health, Centers for Disease Control and Prevention, 1600 Clifton Road NE, Atlanta, GA 30333 USA; 2 Centers for Disease Control and Prevention, Addis Ababa, Ethiopia; 3 Roche Diagnostics, Johannesburg, South Africa; 4 Centers for Disease Control and Prevention, Kigali, Rwanda; 5 Becton Dickinson and Company, Trenton, NJ USA; 6 Africa Centres for Disease Control and Prevention, Addis Ababa, Ethiopia

**Keywords:** Public-private partnership, Viral load, Early infant diagnosis, Laboratory systems strengthening, Cascade

## Abstract

**Background:**

Despite progress towards achieving UNAIDS 90–90-90 goals, barriers persist in laboratory systems in sub-Saharan Africa (SSA) restricting scale up of early infant diagnosis (EID) and viral load (VL) test monitoring of patients on antiretroviral therapy. If these facilities and system challenges persist, they may undermine recorded gains and appropriate management of patients. The aim of this review is to identify Public Private Partnerships (PPP) in SSA that have resolved systemic barriers within the VL and EID treatment cascade and demonstrated impact in the scale up of VL and EID.

**Methods:**

We queried five HIV and TB laboratory databases from 2007 to 2017 for studies related to laboratory system strengthening and PPP. We identified, screened and included PPPs that demonstrated evidence in alleviating known system level barriers to scale up national VL and EID testing programs. PPPs that improved associated systems from the point of viral load test request to the use of the test result for patient management were deemed eligible.

**Results:**

We identified six PPPs collaborations with multiple activities in select countries that are contributing to address challenges to scale up national viral load programs. One of the six PPPs reached 14.5 million patients in remote communities and transported up to 400,000 specimens in a year. Another PPP enabled an unprecedented 94% of specimens to reach national laboratory through improved sample referral network and enabled a cost savings of 62%. Also PPPs reduced cost of reagents and enabled 300,000 tested infants to be enrolled in care as well as reduced turnaround time of reporting results by 50%.

**Conclusions:**

Our review identified the benefits, enabling factors, and associated challenges for public and private sectors to engage in PPPs. PPP contributions to laboratory systems strengthening are a model and present opportunities that can be leveraged to strengthen systems to achieve the UNAIDS 90–90-90 treatment targets for HIV/AIDS. Despite growing emphasis on engaging the private sector as a critical partner to address global disease burden, PPPs that specifically strengthen laboratories, the cornerstone of public health programs, remain largely untapped.

## Background

The Joint United Nations Programme on HIV/AIDS (UNAIDS) fast track treatment targets call for 90% of people living with HIV infection to know their status; 90% of those who know their status to receive antiretroviral therapy (ART); and 90% of those on ART to achieve viral suppression by 2020 [1]. Despite significant progress towards controlling the HIV/AIDS pandemic [2], these new targets have overwhelmed many public health laboratory systems in sub-Saharan Africa (SSA) due to increased demand for early Infant diagnostic (EID) testing of HIV-exposed infants (HEI) and viral load (VL) test monitoring of patients on ART [3]. The VL and EID cascade is characterized by phases from the point of test request to the use of the test results for patient management. There are three phases and include the pre-analytical phase defined as the period from collection of specimens at the referral clinic to receipt of specimens in the laboratory (includes demand creation for testing from care providers and patients, specimen collection and processing, specimen transport system), the analytical phase defined as the period from testing of specimens to obtaining results at the laboratory (includes quality laboratory testing and supply chain management for test reagents and supplies), and the post-analytical phase which entails results transmission from laboratory to receipt of results at the referral clinic and use for patient management (comprised of result reporting to clinics, interpretation and uptake for patient management). Monitoring and evaluation (M&E) is cross-cutting through the different phases and allows monitoring of progress of these phases.

Multiple barriers within the VL and EID cascade prevent optimal access and uptake of test results for better patient management. In the pre-analytical phase, these barriers include lack of patient awareness for available HIV test; non-standardized specimen collection practices, and weak specimen referral networks [4]. In the analytical phase, challenges include frequent equipment breakdown, weak supply chain systems, and unsafe biological waste management. In the post analytical phase, the challenges experienced comprise delayed and inconsistent delivery of test results, limited data systems for reporting results, and poor clinician utilization of laboratory results for improved patient management. There is also a dearth of adequate numbers of competent workforce along all phases of the cascade [4–6].

In 2009 leaders of the US Government’s global AIDS program stated that “The problems we face today will be solved not by governments alone but in partnerships; partnerships with philanthropy, global business and civil societies” [2]. While global PPPs that have improved public health programs such as Global Fund, Foundation for Innovative New Diagnostics, and the Global Alliance for Vaccines and Immunizations (GAVI), have been around for decades [7], PPPs that specifically target the advancement of laboratories, an indispensable component of these programs, are rare. The gap persist despite the increasing emphasis on engaging the private sector as a critical partner to improve services and systems to address diseases that are threats to public health [8–11]. One of the reasons for engagement of fewer than anticipated private entities could be the paucity of empirical data and strategies for effective private sector engagement [12].

Studies suggests that the influx of donor funding has led to decreased private contributions for HIV/AIDS. The reduction in private sector investment and engagement raises concerns about the sustainability of HIV/AIDS programs, particularly in light of the current global economic crisis and emerging competing priorities [13]. There exist opportunities for partnerships to strengthen systems in the VL and EID cascade and address gaps to accelerate achieving the UNAIDS 90–90-90 goals. SSA countries are at different stages of scaling up VL and EID and have varying challenges [5]. Partnerships, especially PPP can play an important role in resolving system barriers affecting VL and EID. In this review, we identify and provide a description of PPPs in SSA that have been used to resolve systemic barriers within the cascade and demonstrated its impact in the scale up of VL and EID.

## Methods

### Data sources and search strategy

We queried five HIV and TB laboratory databases (Medline, Embase, CINAHL, Global Health, Scopus) from 2007 to 2017, with an aim to identify public-private partnerships focused on laboratory system strengthening. We used search terms Public-Private Sector Partnerships, private public partnership, public private partnership, laboratory or specimen handling, specimen transport, specimen referral, sample transport, laboratory quality management, laboratory worker or laboratory technician. Only articles published in English were included.

### Selection criteria

**Fig. 1:**
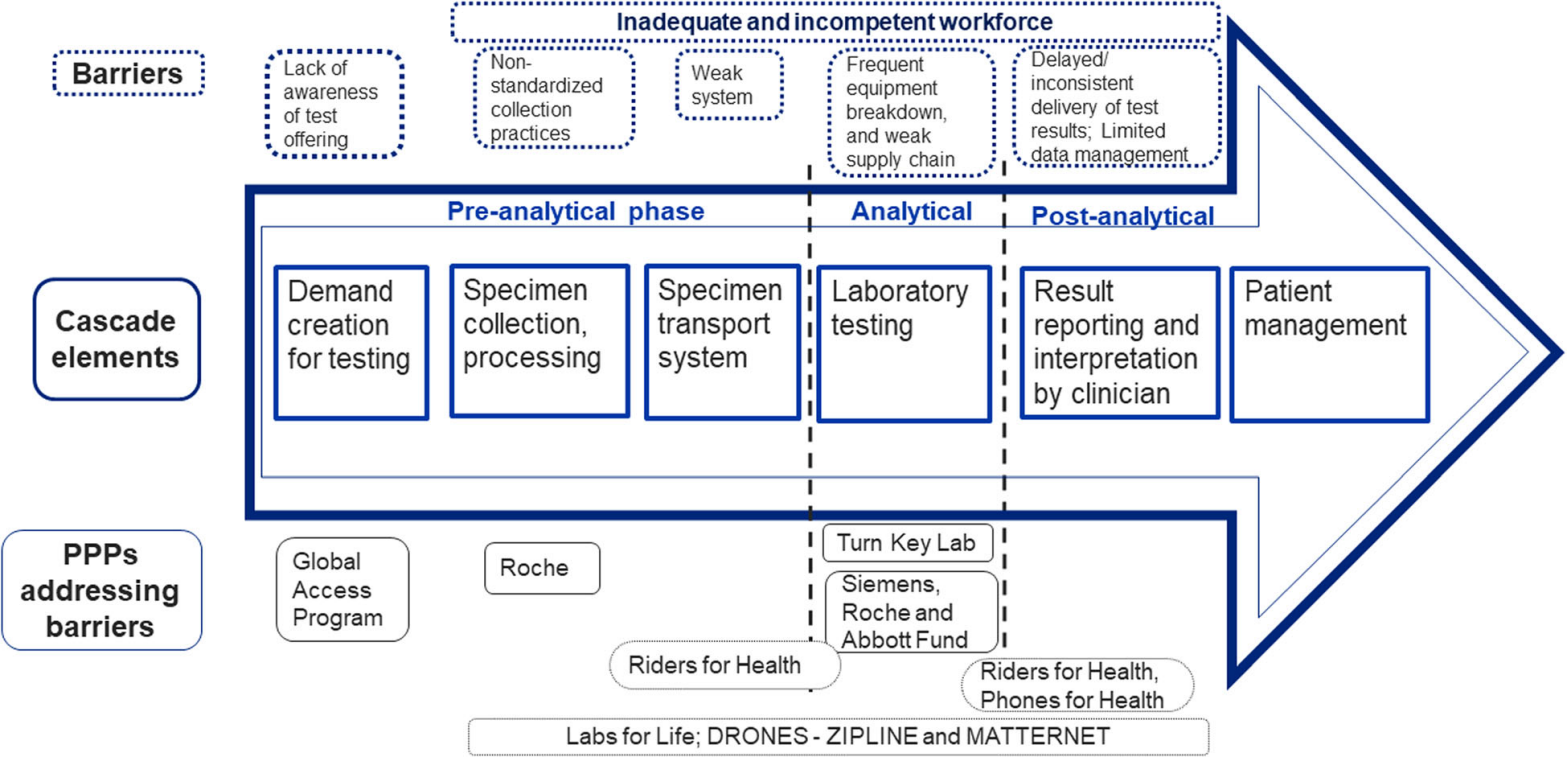
Schematic representation depicting different elements of the viral load and early infant diagnosis cascade (Square boxes within the arrow). The 3 major phases (pre-analytical, analytical, post-analytical) of the cascade are delineated within the arrow. Barriers within the different phases of the cascade are identified above the arrow (dotted line boxes). Public-private partnerships (PPPs) addressing different barrier and at what phase of the cascade have been identified below the arrow (solid line boxes). Pre-analytical phase defined as the period from collection of specimens at the referral clinic to receipt of specimens in the laboratory; the analytical phase defined as the period from testing of specimens to obtaining results at the laboratory and the post-analytical phase entails results transmission from laboratory to receipt of results at the referral clinic and use for patient management. Siemens = PEPFAR is Stronger Together PPP; Turn Key laboratories = PPP between UNITAID, Roche Diagnostics and Clinton Foundation; Labs for Life = PPP between Becton Dickinson and Company and PEPFAR; Roche = PEPFAR's PPP with Roche Diagnostics

We used barriers to scale up VL and EID testing cascade (Fig. 1) as criteria to guide the selection of PPPs in our review. Records of PPPs that were operational in SSA and had successfully implemented interventions to improve systems for demand creation for tests, specimen collection and transportation, laboratory testing, reporting laboratory results to clinics were deemed eligible.

**Fig. 2:**
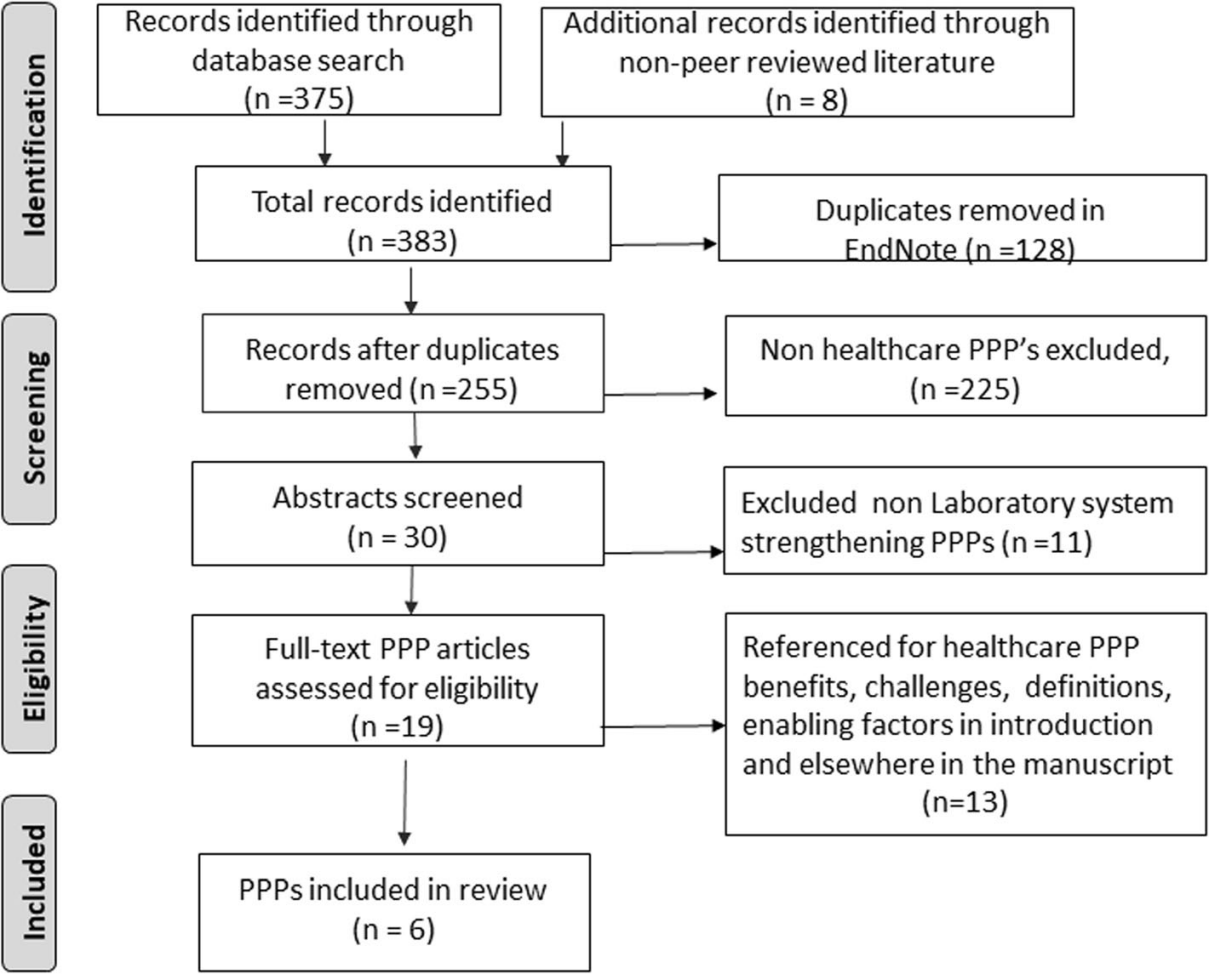
PRISMA flow diagram outlining the different stages of literature review search and selection of Public-Private Partnerships (PPP) in laboratory systems strengthening

A wide spectrum of private sector engagement options exist for delivery of public health programs [14, 15]. PEPFAR defines PPPs as a ‘collaborative endeavors that combines monetary and in-kind resources from the public and private sector to accomplish PEPFAR’s HIV/AIDS prevention, care, and treatment goals’ [16]. All forms of PPP engagements were included in the identification step (Fig. 2).

### Outcomes of interest and data abstraction strategy

Our search yielded 375 records in EndNoteX7 library, and eight from non-peer reviewed sources bringing our total to 383 records (Fig. 2). Using Endnote’s “find duplicates” function we removed 128 duplicates. We systematically assessed each record by evaluating the following broad questions: 1) Was there a clear study objective that addressed barriers to scale up VL and EID testing in record? 2) Did the study use the right methods to address the study objective? 3) Are the results of the study valid? 4) Are the results applicable to my population of interest? Of the 255 remaining articles, 225 records were removed because of their focus on non-healthcare PPPs. We tabulated characteristics of the remaining 30 PPPs by ability of the PPP to address barriers in the various phases of VL and EID cascade, country of operation, name of PPP, start year, intervention type, impact and source using MS Office Excel spreadsheet. Eleven of the 30 abstracts unrelated to laboratory system strengthening were excluded and leaving 19 full text articles for review. Thirteen of the 19 articles provided valuable insights into various modes and interpretations of operationalizing PPPs, benefits and challenges, enabling factors, reasons for dearth of PPPs in global health care. We referenced these 13 articles in introduction and other sections in the manuscript. Six PPP records that met the eligibility criteria were reviewed and analyzed.

## Results

### Pioneering PPPs focused on laboratory system strengthening

Five of the six eligible PPPs have an international scope and the other one is an example of a local PPP. Three of the six PPPs have partnerships with the President’s Emergency Plan for AIDS Relief (PEPFAR) program and the other two are independent of PEPFAR (Table 1).

**Table 1:** Summary of Public-Private Partnerships (PPPs) (2007–2017) that address barriers and strengthen laboratory systems in resource-limited settings to improve access, coverage, quality and utilization of Viral Load and Early Infant Diagnosis testing

Cascade phase	Barrier	Country	PPP	PPP Intervention	Impact	Source
Pre-analytical Phase	Poor and non-standardized specimen collection procedures.	Kenya	L4L	• Trained 91 HCW on safe phlebotomy collection practices.	• Increased knowledge of phlebotomists by 41%. • Integration of safe phlebotomy practices into pre-service training.	Kimani et.al., [26]
	Weak supply chain and unreliable specimen transportation system.	Gambia, Kenya, Lesotho, Malawi, Nigeria, Zambia, Zimbabwe.	Riders for Health	• Accessed hard-to- reach communities for healthcare needs by providing motorcycles for transportation. • Trained healthcare workers on managing supply chain distribution of medicines, transportation of specimens and return of results and managed emergency referrals.	• Improved access to 14.5 million people to healthcare. • Transported 400, 000 specimens/year between laboratory and healthcare facilities.	WHO [23, 28] World Bank [15]
	Weak specimen transportation system.	Uganda	L4L	• Use of GIS to map efficient sample referral network. • Provided standardized specimen transportation materials. • Training of transporters to safely package and transport specimens.	• Ten-fold increase in referrals of patients sample with presumptive MDR-TB. • 94% specimens reached the national laboratory within the established target time of 72 h.	Joloba et al., [27]
Analytical Phase	Lack of skilled workforce, modern laboratory infrastructure to provide timely and accurate services to patients.	SSA	Global Access Program	• Engaged manufacturer and negotiated lower prices for HIV VL and EID reagents.	• 300, 000 infants enrolled into care and treatment. • Provided 900,000 tests for EID. • – Projected anticipated cost savings of $150 million in next 5 years.	Roche Diagnostics [20]
		SSA	Turn Key Laboratory	• Set up‘Turn Key Laboratory’ for access to pediatric testing.	• 900,000 tests were made available. • 100 laboratories in SSA now routinely offer PCR for EID.	Roche Diagnostics [20]
		Mozambique	L4L	• Establishment of national laboratory quality assurance program to facilitate stepwise quality improvement of laboratory services.	• Trained and mentorship resulted in 18 MOH qualified auditors and 28 manager/quality officers capacitated to manage improvements of laboratories and steer towards accreditation.	Skaggs et al., [29]
		Tanzania	Abbott Fund	• Built and modernized 23 regional-level laboratories, • Built outpatient center at the national hospital serving 1000 patients/day. • Provided mentorship.	• 10 fold increase (from 110,000 to 1,158,000) in test volumes in 5 years. • Improved healthcare services for people living with HIV and other chronic diseases across the country.	Abbott Fund [25]
Post - analytical phase	Delayed and inconsistent delivery of VL and EID test results to patients.	Ethiopia	L4L	• Used GIS to map and network 554 clinic facilities to laboratories testing for VL, EID, CD4 and hematology. • Procured 400 standard specimen transportation containers. • Trained 586 and 81 laboratory and postal workers, respectively.	• 50% reduction in TAT (from specimen collection to reporting results) for ART patients (10 to 5 days). • Standardized training module used for training in all the regions • 62% in cost savings for transporting EID specimens. • Reduced TAT from 1 to 2 months to 5–10 days.	Kebede et al., [32] Kiyaga et al. [6]
		Kenya, Tanzania and Rwanda	Phones for Health	• Allowed input of health data and transfer to central database. • Enabled ordering medicines, sending alerts and download of guidelines. • Enabled access to training materials. • Facilitated transmission of results to SMS printers.	• Improved access to knowledge and information of 50,000 community health workers. • Reduced TAT for results delivery • Effective monitoring of mother-to-child transmission through EID systems rolled out to 63 sites nationally.	UNAIDS [25], Fogarty [24]

a = Labs for Life;

Abbreviations: *L4L* Labs for Life, *HCW* healthcare workers, *GIS* geographic information system, *MDR-TB* multidrug resistant tuberculosis, *PCR* polymerase chain reaction, *SSA* sub Saharan Africa, *VL* viral load, *EID* early infant diagnosis, *ART* antiretroviral therapy, *MOH* ministry of health, *TAT* turnaround time, *WHO* World Health Organization, *CD4* cluster of differentiation 4, *SMS* short message service

Memoranda of Understanding (MOUs) were signed between PEPFAR and three private companies to focus on strengthening laboratory networks and systems to support ART scale up. The MOUs were signed between the US Centers for Disease Control and Prevention (CDC), Office of Global AIDS Coordinator—PEPFAR implementing office and three private companies: Becton Dickinson and Company (BD) [17]; Roche Diagnostics [18] and Siemens Healthineers [19] for $18, $10 and $15 million, respectively, in shared resources. In 2012, BD and PEPFAR renamed the partnership, Labs for Life, and renewed it for $20 (2012–2017) and $12 (2018–2020) million, respectively.

i). **Labs for Life** (L4L) strengthened access to ART by building standardized in-country capacity for specimen collection, referral and result reporting systems. With operations in Uganda, Ethiopia, Mozambique, South Africa, Kenya and India, BD deployed experts to provide in-country training (Fig. 1 and Table 1).
ii). **Roche** has three PPPs designated for the VL and EID programs. In 2008, only 15% of HEI in SSA were accessing EID services during the first two months of life [20]. Roche Diagnostics responded to this critical service gap with a PPP agreement with UNITAID and the Clinton HIV/AIDS Initiative (CHAI). Roche provided molecular diagnostics supplies and set up ‘Turn Key Laboratories’, to provide timely HIV testing for the pediatric population [20]. In 2012, Roche and PEPFAR partnered to create capacity for a well-trained laboratory cadre offering didactic courses at the Roche Scientific Campus (RSC) in Johannesburg, South Africa. In **2014,** Roche signed another landmark PPP, known as the Global Access Program, which negotiated and lowered the price of VL tests in low- and middle-income countries (Fig. 1 and Table 1).
iii). **Stronger Together** is a five-year PPP that was signed in 2014 between PEPFAR and Siemens Healthineers, the new brand name of Siemens Healthcare company. The goal of ‘Stronger Together’ was to develop a competent laboratory workforce globally through a virtual education platform on social media [21].
iv). **Abbott Fund,** a global healthcare company, demonstrated a unique example of a locally operated PPP in Tanzania in response to the growing HIV epidemic to rapidly scale up HIV care and treatment activities. In 2001, Ministry of Health and Social Welfare (MOHSW) signed a PPP with Abbott Fund for expanded access to health care and to strengthen laboratory infrastructure [22] and system capacity (Fig. 1 and Table 1).
v). **Riders for Health** PPP was established 25 years ago in the United Kingdom, and continues now to be managed out of Africa [15] to address lack of access to health care among residents in remote communities in seven countries. Riders for Health managed a fleet of 1300 motor cycles and a variety of four wheeled vehicles in harsh conditions with little infrastructure to connect vital health care with rural areas hitherto unreachable except on foot [23].
vi). **Phones for Health** PPP was initiated in 2007 with operations in countries including Kenya, Tanzania, and Rwanda, leveraging over $3 million annually [24]. Utilizing mobile phone technologies or mHealth as tools and platforms for health research and healthcare delivery [25]. It is a PPP between the health care software provider Voxiva, the phone producer Motorola, the telecom company MTN, the GSMA Development Fund, PEPFAR, CDC Foundation and Accenture Development Partnerships.

### Impact of PPPs addressing barriers to scale up HIV viral load and early infant diagnostic testing

Taken together, the collective contributions of the six eligible PPPs have impacted a cost savings of 62%, increased access to 14.5 million patients to healthcare, transported 400,000 specimens/year, tested 300,000 additional HEI, reduced turnaround time of reporting results by 50%. The specific contributions of the six PPPs within the pre-analytical, analytical and post analytical phases of the VL and EID cascade are highlighted below (Fig. 1 and Table 1).

A. Pre-analytical phase

i). **Demand creation for testing:** In 2014, when Global Access Program lowered the price of VL testing by 40% thereby increasing its affordability and availability, it was projected that this PPP will save more than $150 million in costs over the next 5 years [20].
ii). **Specimen collection and processing:** L4L PPP made significant progress in improving blood draw practices by developing and institutionalized a curriculum for phlebotomists to standardize safe blood-drawing procedures in Kenya. Following the initial curriculum training, the average knowledge increase was 41% for phlebotomists [26], which they went on to apply in their practices.
iii). **Specimen transport system**: In 2007, Uganda’s effort to control the spread of the deadly Multidrug-Resistant Tuberculosis (MDR-TB) was severely limited by weak laboratory network coupled with inadequate specimen referral and result reporting system. L4L PPP collaborated with Ugandan Ministry of Health (MOH), CDC and local partners to use geographic information system (GIS) technology to map and strengthen a national specimen referral system [27]. This resulted in 94% of specimens reaching the national laboratory within the established target time of 72 h. This model later served as the cornerstone for the EID specimen transportation and result reporting system and generated a cost savings of 62% and reduced average turnaround time from 1 to 2 months to 5–10 days [6]. Riders for Health transported 400,000 blood and sputum samples between laboratories and health centers every year and improved access to healthcare for 14.5 million people in Gambia, Kenya, Lesotho, Malawi, Nigeria, Zambia, Zimbabwe [15, 28].

B. Analytical phase

i). **Laboratory testing:** Roche’s PPP for “Turn Key Laboratory” introduced a paradigm shift for expanding laboratory services for pediatric HIV patients resulting in 900,000 tests being made available, an increase of 300,000 infants enrolled into care and treatment, and Polymerase Chain Reaction (PCR) testing being routinely provided in more than 100 laboratories [20].
ii). L4L PPP has also played a significant role in strengthening Continuous Quality Improvement (CQI) of laboratories in several SSA countries including Mozambique, Kenya, Ethiopia, India. In a joint collaboration, L4L helped MOH Mozambique program by providing training and mentorship that resulted in 18 MOH qualified auditors and 28 manager/quality officers capacitated to establish a national laboratory quality assurance program [29].
iii). In collaboration with CDC Tanzania and partners such as Design 4 Others and the Association of Public Health Laboratories, the Abbott Fund’s PPP contributed $10 million to building and strengthening a network of 23 regional-level laboratories in Tanzania [25]. These laboratories provided support for 120 district hospital laboratories resulting in improved healthcare services. Abbott Fund provided mentoring, technical support, and expertise in the areas of construction, engineering, infection control, waste management, information technology, and laboratory management [30]. This PPP also built a modern outpatient center at the Muhimbili National Hospital serving over 1000 patients a day for HIV and non-communicable ailments and the system strengthening efforts increased by 10 fold laboratory tests volumes [31].

C. Post analytical phase

i). **Result reporting and interpretation by clinicians:** From 2010 to 2012, L4L, MOH and CDC partnership in Ethiopia reduced the turnaround time of laboratory patient results by 50% from 10 to 5 days [32] in 554 (59%) of the 944 districts. The program has demonstrated sustainable expansion covering 800 (85%) of the 944 districts as of 2017 and transports specimens for CD4, EID, chemistry, hematology and TB tests, independent of the PPP.
ii). Phones for Health PPP enabled health workers to input health data and transfer them to a central database, order medicines, send alerts, download guidelines, and access training materials. In Rwanda, it empowered practitioners to monitor antiretroviral drug stocks in real time, and accelerated the return of CD4 and viral load test results to remote facilities. In September 2012, 252 of the 457 health facilities were using the electronic system (> 50% of coverage) [33]. Kenya and other countries have used the mhealth technology to transmit laboratory results to mobile phones which are sent to SMS printers in referring clinics to utilize for patient management [24, 25, 34–36].

## Discussions

### Opportunities for PPPs to scale up HIV viral load and early infant diagnostic testing

Globally, as of 2016, 70% of people living with HIV (PLHIV) were diagnosed, 77% of those were on ART, and 82% of those were virally suppressed [37]. This finding highlights the need for additional synergistic partnerships to close the gap in HIV testing, treatment, and viral suppression. Since 2006, the U.S. has been engaged in nearly 300 partnerships, which have yielded nearly $400 million in private sector investment and $335 million in funding from PEPFAR [38]. More PPP are needed with a focus to strengthen laboratory systems and networks. There are still challenges, for instance, access to testing, return of laboratory test results to patients, ensuring quality of testing, develop a cadre of trained workforce, and present opportunities for collaborations to increase efficiencies. PPPs can play a key role in addressing some of these challenges.

- **Uptake of VL and EID test results:** To close the knowledge gap of clinicians (e.g., nurses, midwives, clinical officers, medical doctors) for the uptake of viral load test results [39], members of African Regional Collaborative for Laboratory Technologists collaborated with Roche-PEPFAR PPP and provided VL training to nurse leaders from 17 countries in SSA. The training emphasized the role of nurses in initiating request for VL testing and utilizing VL test results for patient management in ART clinics [40].
- **Safe and standardized specimen collection process:** Phlebotomy-related errors account for > 60% of errors in the pre-analytical phase [41] and can lead to delayed or incorrect results, with unjustifiable costs to patient. Roche-PEPFAR PPP collaborated with the US CDC to develop an online training tool to standardize specimen collection procedures using an alternate specimen type - dried blood spots (DBS), to improve access to viral load testing. The training is accessible at the African Society for Laboratory Medicine (ASLM) portal in English, French and Portuguese [42].
- **Quality assured test results to clients:** Strengthening the tiered laboratory network in a country to expand and ensure access to reliable, high-quality VL and EID testing services is critical [39]. US CDC developed a CQI program and Roche-PEPFAR PPP continue to offer the CQI program in its campus in Johannesburg. Average pretest scores in five courses for 94 students from 22 countries rose from 12 to 88% (personal communication, facilitator Anna Murphy, affiliation ASLM). To develop a sustainable, cost-effective solution for wider dissemination, Stronger Together PPP replicated the two week long didactic curriculum into an e-library of 48 training videos, available free of cost through an innovative e-platform [43]. This approach has caught the interest of MOH in several African countries and can also be used to deploy training materials in any area including VL and EID.
- **Specimen transport and result reporting system:** Innovative strategies such as unmanned aerial vehicles to pick up specimens and deliver results within Switzerland (Matternet company) [44] and in Rwanda and Tanzania (Zipline company) [45] are examples of technology for use in improving laboratory-clinic interface. These innovations could be leveraged to improve delivery of VL and EID results in similar contexts.

Other unmet needs in the VL cascade represent opportunities for new partnerships. The needs are inadequate and competent workforce, unsafe disposal of hazardous waste materials generated due to increased volume of testing, equipment maintenance, limited data management options to improve laboratory-clinic interface (Fig. 1).

### Benefits of PPP to private and public sector

PPPs provided an avenue for private entities to gain access and improved understanding of government’s policies and strategies, which can increase market knowledge and awareness of national priorities. By engaging in PPPs, the private sector can also benefit the local workforce by providing increased access to resources; introducing new goods and services; sharing product/service risks and investments [7], identifying and increasing local expertise; institutionalizing interventions; and build sustainable laboratory networks with regard to public-supported laboratories [32]. From the public sector perspective, effective PPPs provide additional capabilities, flexibility, skills, resources and funding, which enhance their ability to respond to the demands for increased services or scale-up programs of national importance. Furthermore, at the country-level, PPP’s collaboration can empower countries to mobilize funds and direct them towards highest priority activities; facilitate research and development; and improve affordable healthcare interventions [12].

### Strengths and limitations

This review successfully identified PPPs that have contributed to achieving UNAIDS 90–90-90 treatment goals. While most of the PPP were international, we observed the important role played by local indigenous PPP and further highlighting the important contribution of PPPs. We are cognizant of the small number of published studies highlighting PPP in laboratory system strengthening. Nonetheless, their impact remains unquestionable and underscore the need for more laboratory systems and service delivery focused PPP.

## Conclusions

Clearly PPP plays a critical role towards achieving the UNAIDS 90–90-90 targets. Despite the growing emphasis on engaging the private sector as a critical partner to address health care systems, PPPs that strengthen laboratories, the cornerstone of public health programs remain largely untapped. Increased partnerships between public sector and private companies are needed to synergistically address the challenges of achieving universal HIV testing, VL and EID scale up. Opportunities for new local and global PPPs should be harnessed for a standardized and sustained scale up of VL and EID to reach the 90–90-90 goals.
